# Low diversity or poorly explored? Mesophotic molluscs highlight undersampling in the Eastern Mediterranean

**DOI:** 10.1007/s10531-020-02063-w

**Published:** 2020-10-12

**Authors:** Paolo G. Albano, Michele Azzarone, Bruno Amati, Cesare Bogi, Bruno Sabelli, Gil Rilov

**Affiliations:** 1 Department of Palaeontology, University of Vienna, Althanstrasse 14, 1090 Vienna, Austria; 2 Largo Giuseppe Veratti, 37/D, 00146 Rome, Italy; 3 Gruppo Malacologico Livornese, c/o Museo di Storia Naturale del Mediterraneo, via Roma 234, 57127 Livorno, Italy; 4 Museo di Zoologia dell’Università di Bologna, via Selmi 3, 40126 Bologna, Italy; 5 National Institute of Oceanography, Israel Oceanographic and Limnological Research (IOLR), 3108001 Haifa, Israel

**Keywords:** Mollusca, Mesophotic zone, Twilight zone, Sponge reefs, Levantine Basin

## Abstract

Mesophotic assemblages are the next frontier of marine exploration in the Mediterranean Sea. Located below recreational scuba diving depths, they are difficult to access but host a diverse array of habitats structured by large invertebrate species. The Eastern Mediterranean has been much less explored than the western part of the basin and its mesophotic habitats are virtually unknown. We here describe two mesophotic (77–92 m depth) molluscan assemblages at a rocky reef and on a soft substrate off northern Israel. We record 172 species, of which 43 (25%) are first records for Israel and increase its overall marine molluscan diversity by 7%. Only five of these species have been reported in recent surveys of the nearby Lebanon, suggesting that our results are robust at a broader scale than our study area and that the reported west-to-east declining diversity gradient in the Mediterranean needs a reappraisal based on proper sampling of the eastern basin. We found only four (2%) non-indigenous species, represented by seven (0.5%) specimens. These results suggest that pristine native assemblages still thrive at this depth in Israel, in contrast to the shallow subtidal heavily affected by global warming and biological invasions, calling for strong conservation actions for these valuable but vulnerable habitats.

**Electronic supplementary material:**

The online version of this article (10.1007/s10531-020-02063-w) contains supplementary material, which is available to authorized users.

## Introduction

Mesophotic ecosystems are located between ~ 40 m and ~ 150 m depth where low light is a dominant abiotic feature (Lesser et al. 2009; Cerrano et al. 2010). Initially defined in tropical areas, this depth zone is increasingly being explored in the Mediterranean Sea uncovering a diverse array of communities dominated by algae (Ballesteros 2006; Bo et al. 2011; Joher et al. 2012), anthozoans (Bo et al. 2009, 2011; Cerrano et al. 2010; Costantini and Abbiati 2016; Grinyó et al. 2016; Corriero et al. 2019; Chimienti et al. 2020), bivalves (Cardone et al. 2020) and sponges (Bo et al. 2011; Idan et al. 2018). Notwithstanding the depth, these communities are under anthropogenic pressure due to stressors such as climate warming (Cerrano et al. 2000; Garrabou et al. 2001, 2009), fishing and trawling (Rossi 2013; Bo et al. 2014). Little is still known about their biodiversity: due to the difficulties of access, most of the surveys focused on the large dominant species. Additionally, with very few exceptions (e.g. Idan et al. 2018) research has been conducted mainly in the western Mediterranean Sea: eastern Mediterranean mesophotic habitats are virtually unknown.

Molluscs are a taxonomically and functionally diverse phylum and some species can be important ecosystem engineers (Gutiérrez et al. 2003). They show a high level of correlation with overall species richness and community patterns on both hard and soft substrates (Ellingsen 2002; Smith 2005). They thrive in all marine habitats, but most species prefer micro-habitats such as crevices, the underneath of stones, plant/algal assemblages or to live infaunally to escape predation (Beesley and Ross 1998; Albano and Sabelli 2012). Molluscs do not represent an exception to the general limited knowledge of the Eastern Mediterranean Sea. Although some work on native assemblages is available, research has been polarized on the burgeoning number of non-indigenous species, here mostly of Red Sea origin (e.g. Mienis 2010; Albayrak 2011; Crocetta et al. 2017; Steger et al. 2018). Importantly, no work has been conducted on mesophotic molluscs.

We describe the results of recent surveys on two mesophotic (77–92 m depth) assemblages off northern Israel: a rocky reef and a soft substrate. We recorded 172 species, of which 43 (25%) are first records from Israel. Only four species were non-indigenous and suggest that pristine native assemblages still thrive at this depth, in contrast to the shallow subtidal, calling for strong conservation actions for these valuable but vulnerable habitats.

## Materials and methods

**Fig. 1:**
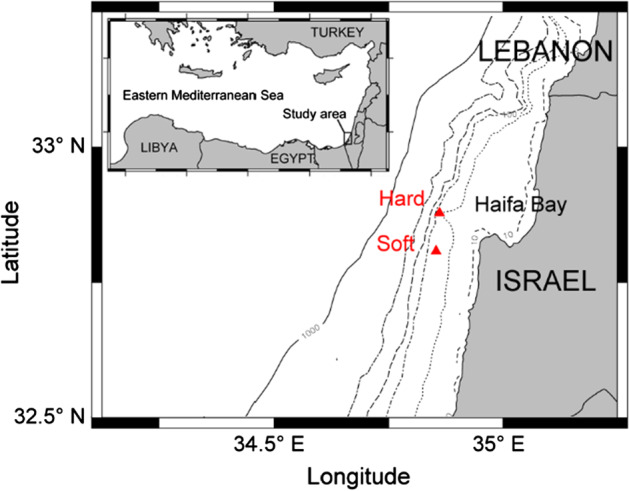
Position of the studied localities off northern Israel, Eastern Mediterranean Sea

**Fig. 2:**
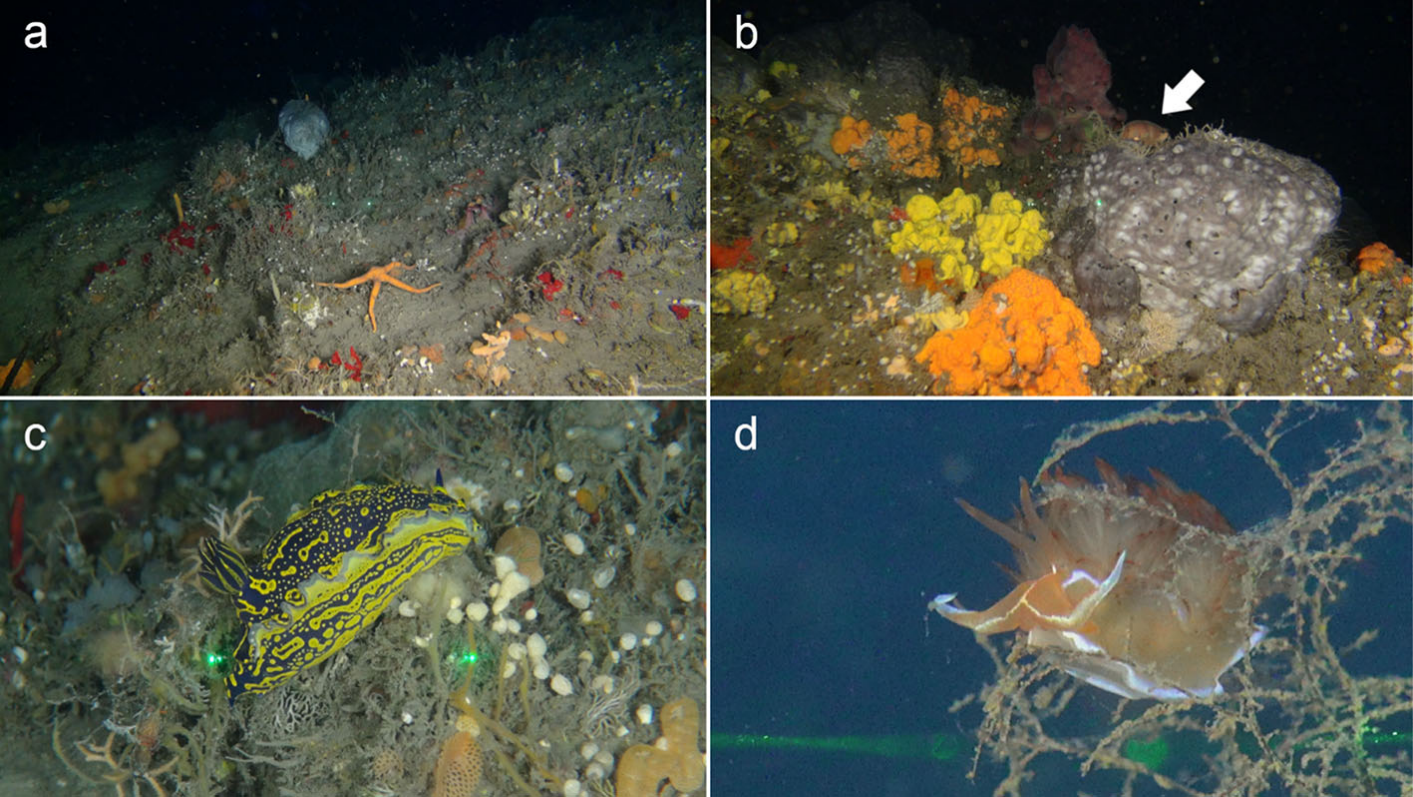
**a–b** Sponge dominated reef off Rosh Carmel, Israel, at 92 m depth. The arrow in panel **b** indicates the living gastropod *Luria lurida*. **c** The nudibranch *Felimare picta*. **d** The nudibranch *Dondice banyulensis* (Photo credits: EcoOcean)

Sampling was conducted at two localities off northern Israel with different substrates: a rocky reef near the western tip of the Rosh Carmel plateau and a soft substrate off Atlit (Fig. 1; Table 1). The rocky reef lies at 92 m depth and had a sponge cover of ~ 40% (Fig. 2). It was sampled at 12 stations with a rock dredge: a steel tube of 20 cm of diameter, provided with a net of mesh size 0.5 cm. The soft substrate was a muddy bottom at 77–83 m depth and was sampled with a Van Veen grab (36.5 × 31.8 cm) collecting three replicates. Samples from both substrates were sieved with a 0.5 mm mesh and fixed in ethanol. Live collected individuals and empty shells were picked in the lab, and stored in ethanol or air dried, respectively. A checklist of the native species of Israel was compiled from the literature: since the first record of a species by Weinkauff (1867), 624 species have been recorded. A screening of the literature shows that 572 species occur in the mesophotic zone (40–150 m). The systematic arrangement follows Bouchet et al. (2017) for the gastropods, Bouchet et al. (2010) for the bivalves and Steiner and Kabat (2001) for the scaphopods. Species authorship is provided in the ESM Table S1.

**Table 1:** List of sampling stations

Station	Locality	Latitude [°N]	Longitude [˚°]	Depth [m]	Date	Device	Substrate	
Hard substrate samples								
CH2, RC6, RC16, RC27, RC33, RC36, RC44, RC50, RC51, RC60, RC61, RC66	Rosh Carmel	32.8779	34.8612	92	25/06/2018	Grab	Rocks	
Soft substrate samples								
TG80_1,TG80_2,TG80_3	Off Atlit	32.80770	34.85371	77–83	21/09/2016	Grab	Mud	

Sample coverage (completeness) and asymptotic diversity at perfect coverage (= 1) were computed with the iNEXT package after pooling living and dead occurrences per type of substrate (Chao and Jost 2012; Hsieh et al. 2016). The map was drawn with the oceanmap R package.

Shell photographs were taken using a Zeiss SteREO Discovery.V20 stereomicroscope and stacked with Helicon Focus 6, and with a Fei Inspect S50 scanning electron microscope without coating.

## Results

**Fig. 3:**
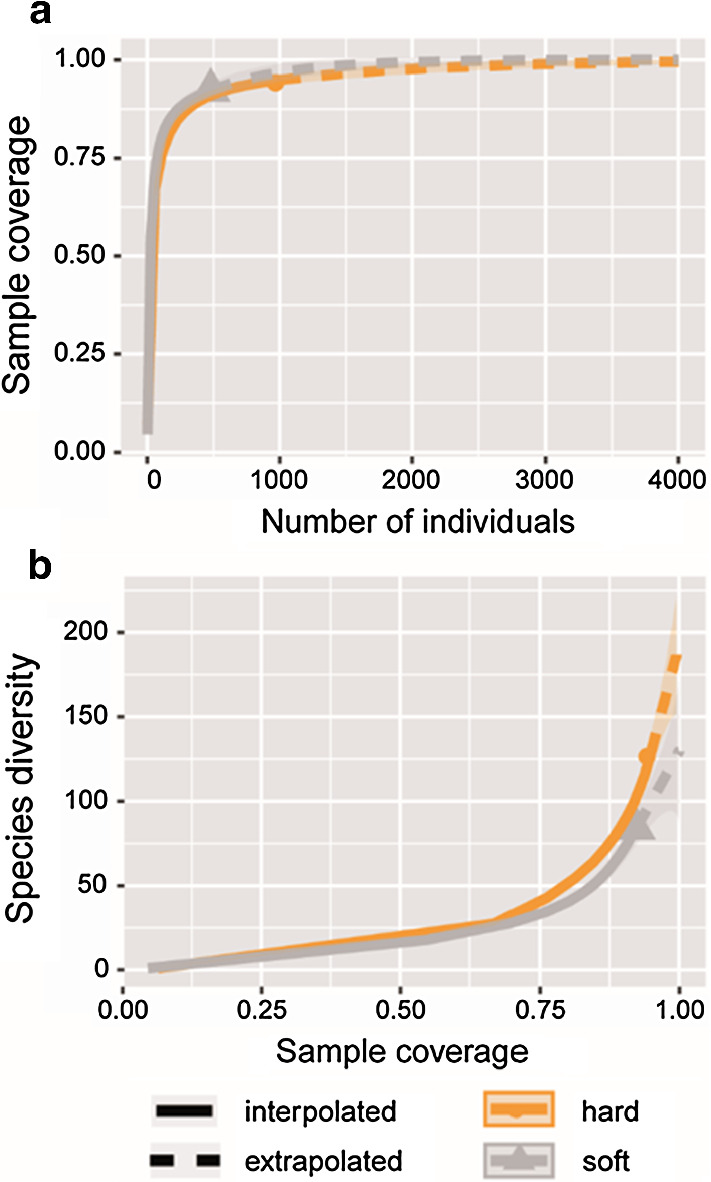
Sample completeness (coverage) and estimated richness at perfect coverage at two mesophotic sites off northern Israel. **a** Sample coverage is very high reaching 94.5% and 92.3% on hard and soft substrates, respectively. **b** Notwithstanding the high coverage, the extrapolated diversity at perfect coverage is 193 (+ 51%) and 130 (+ 55%) species on hard and soft substrates, respectively

We found 135 and 6 living individuals, and 865 and 477 empty shells, on hard and soft substrates, respectively, belonging to 131 and 87 species, for a total of 172 species (ESM Table S1). Sample coverage was very high: 94.5% and 92.3% on hard and soft substrates, respectively, but still, the extrapolated diversity at perfect coverage (= 1) was 193 (+ 51%) and 130 (+ 55%) species (Fig. 3).

**Fig. 4:**
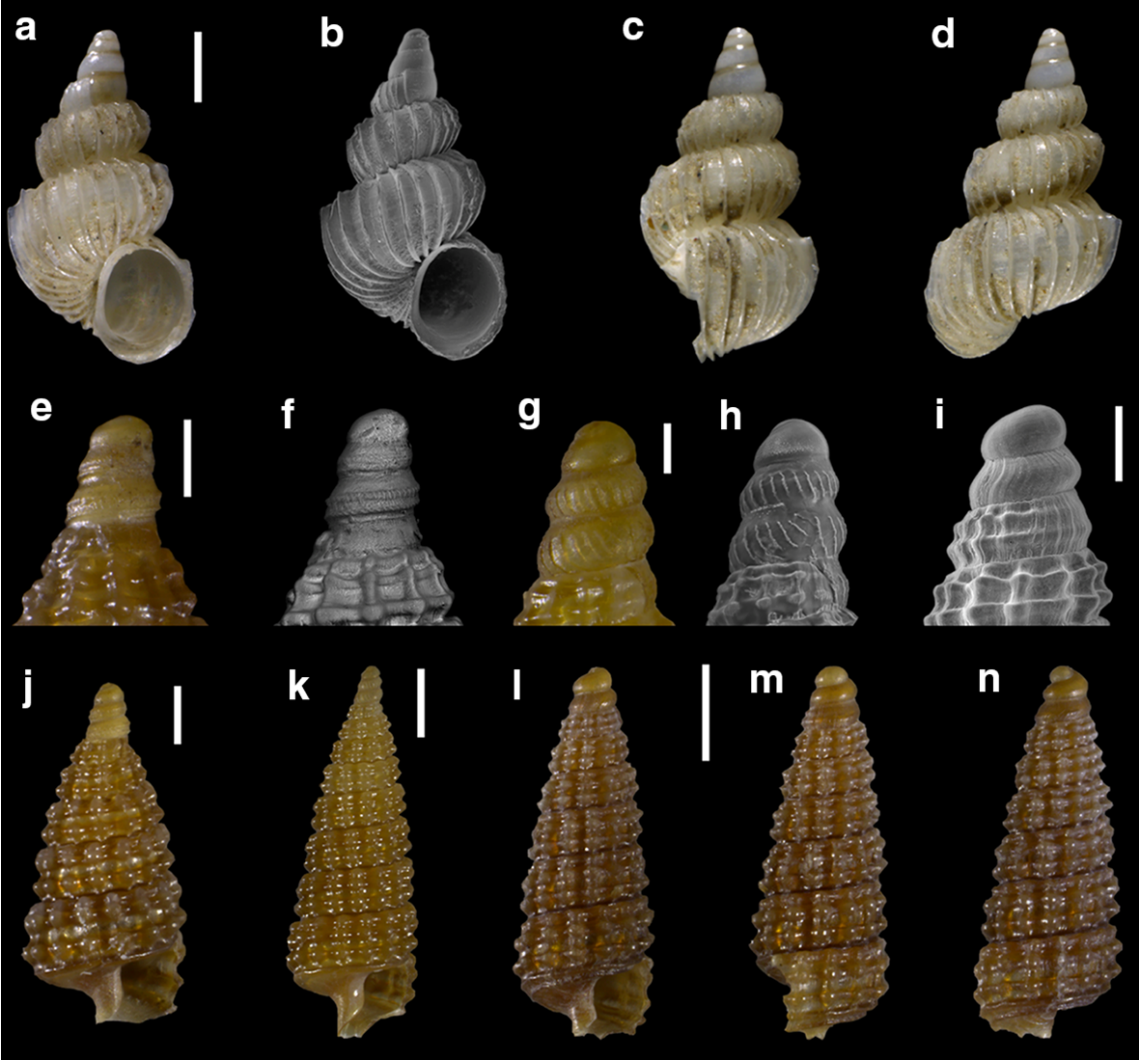
**a–d** *Epitonium tryoni*, sample TG80. **e–f, j** *Cerithiopsis pulchresculpta*, sample RC16. **g–h, k** *Cerithiopsis scalaris*, sample RC16. **i**, **l–n** *Krachia cylindrata*, sample RC16. Scale bars: a–d, I: 0.2 mm; e–h: 0.1 mm; j: 0.2 mm; k–n: 0.5 mm

**Fig. 5:**
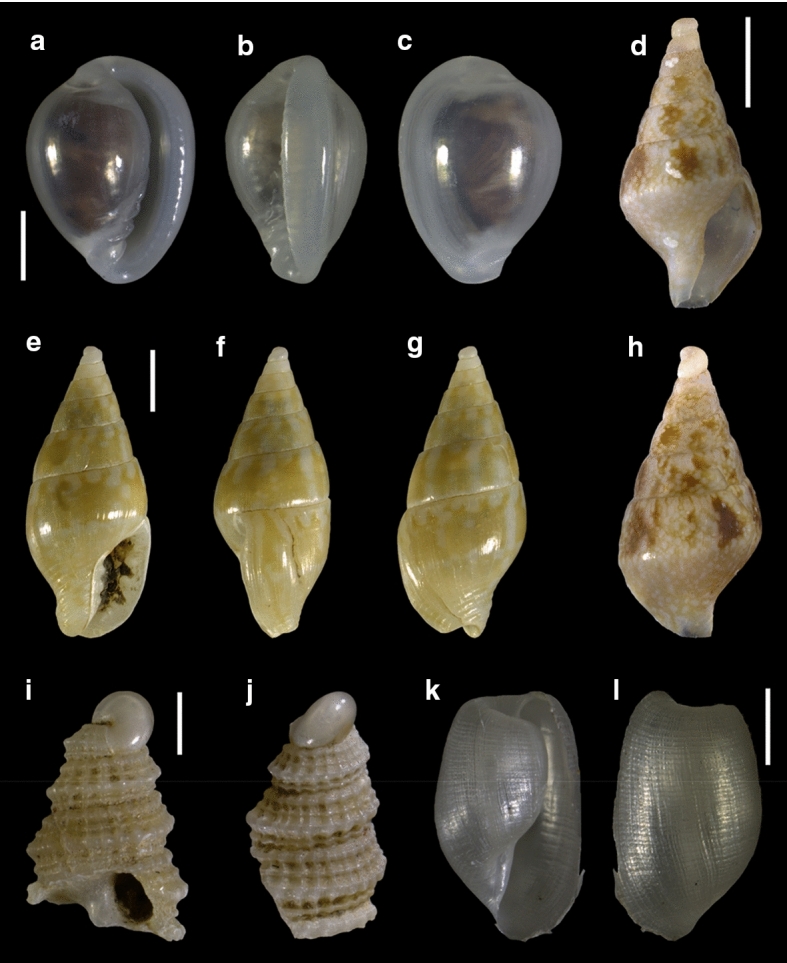
**a–c** *Granulina melitensis*, sample TG80. **d**, **h** *Mitrella svelta*, sample RC16. **e–g** *Mitrella coccinea*, sample RC16. **i–j** *Mathilda bieleri*, sample RC16. **k–l** *Notodiaphana atlantica*, sample RC51. Scale bars: a–c, i–l: 0.5 mm; d–h: 2 mm

**Fig. 6:**
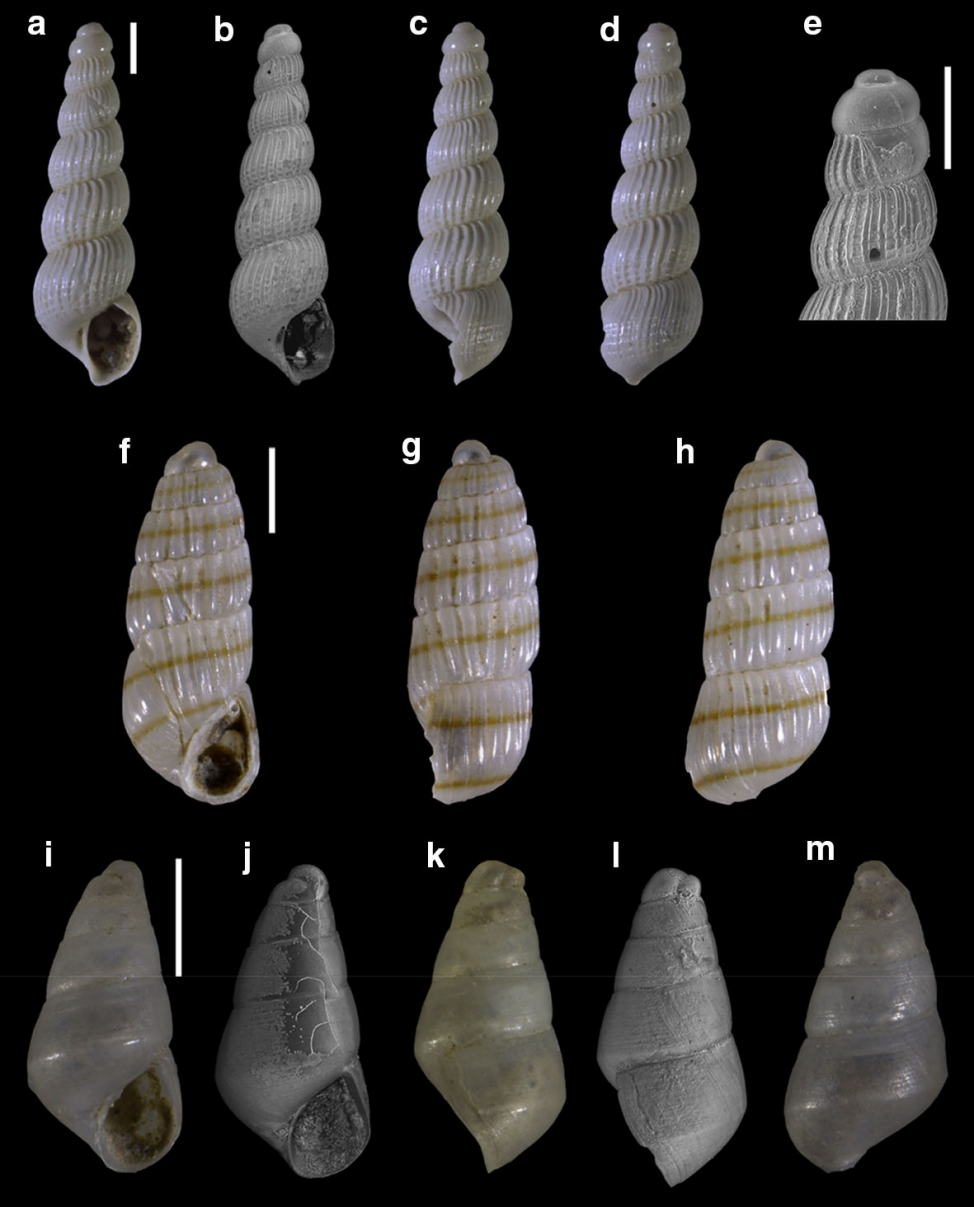
**a–e** *Graphis albida*, sample TG80. **f–h** *Odostomella bicincta*, sample RC16. **i–m** *Odostomia turrita*, sample RC16. Scale bars: a–e: 0.2 mm; f–m: 0.5 mm

**Fig. 7:**
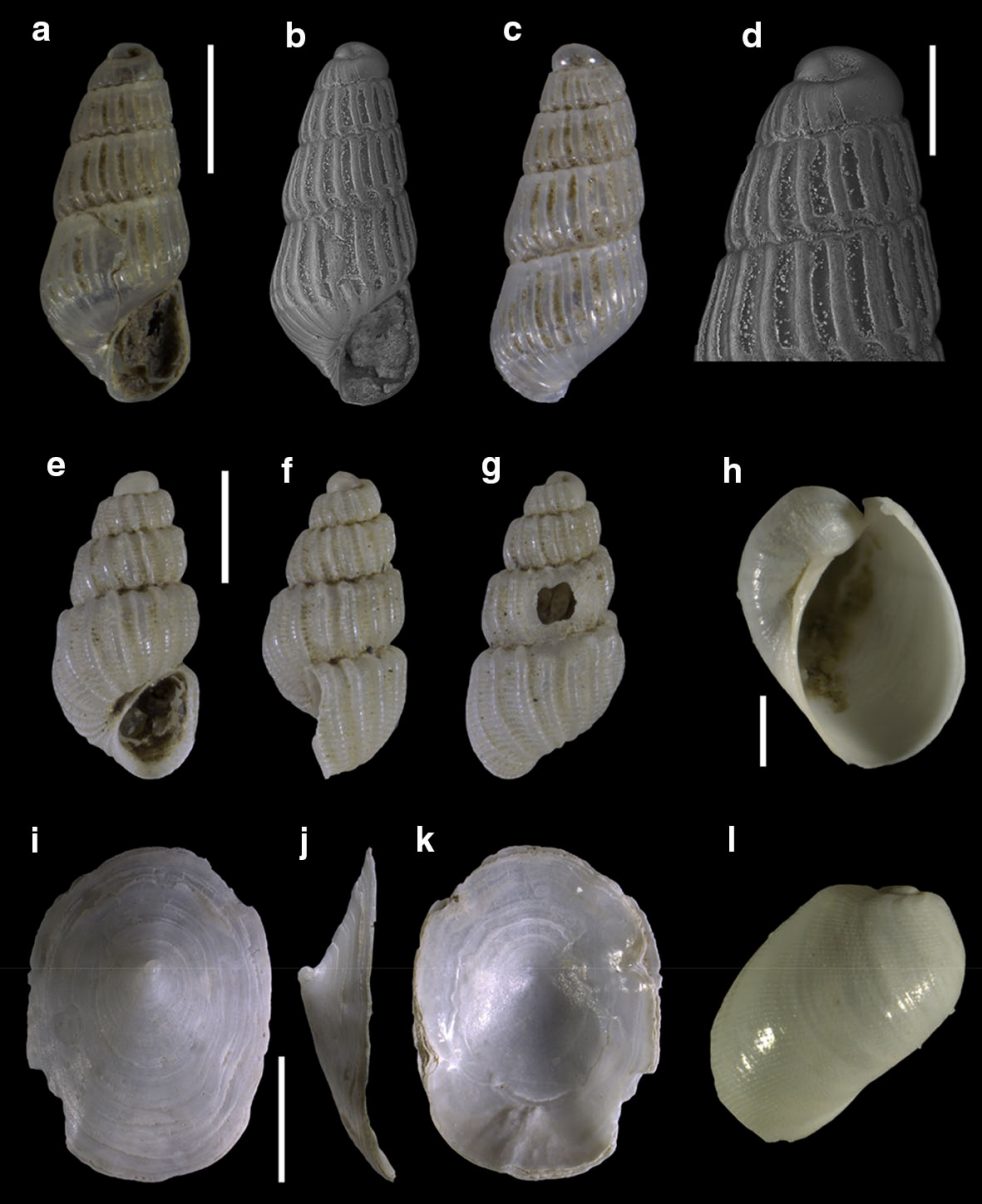
**a–d** *Parthenina penchynati*, sample RC16. **e–g** *Pyrgulina stefanisi*, sample TG80. **h**, **l** *Philine punctata*, sample TG80. **i–k** *Tylodina perversa*. Scale bars: a–c: 0.5 mm; d: 0.2 mm; e–g: 0.5 mm; h, l: 0.2 mm; i–k: 2 mm

**Fig. 8:**
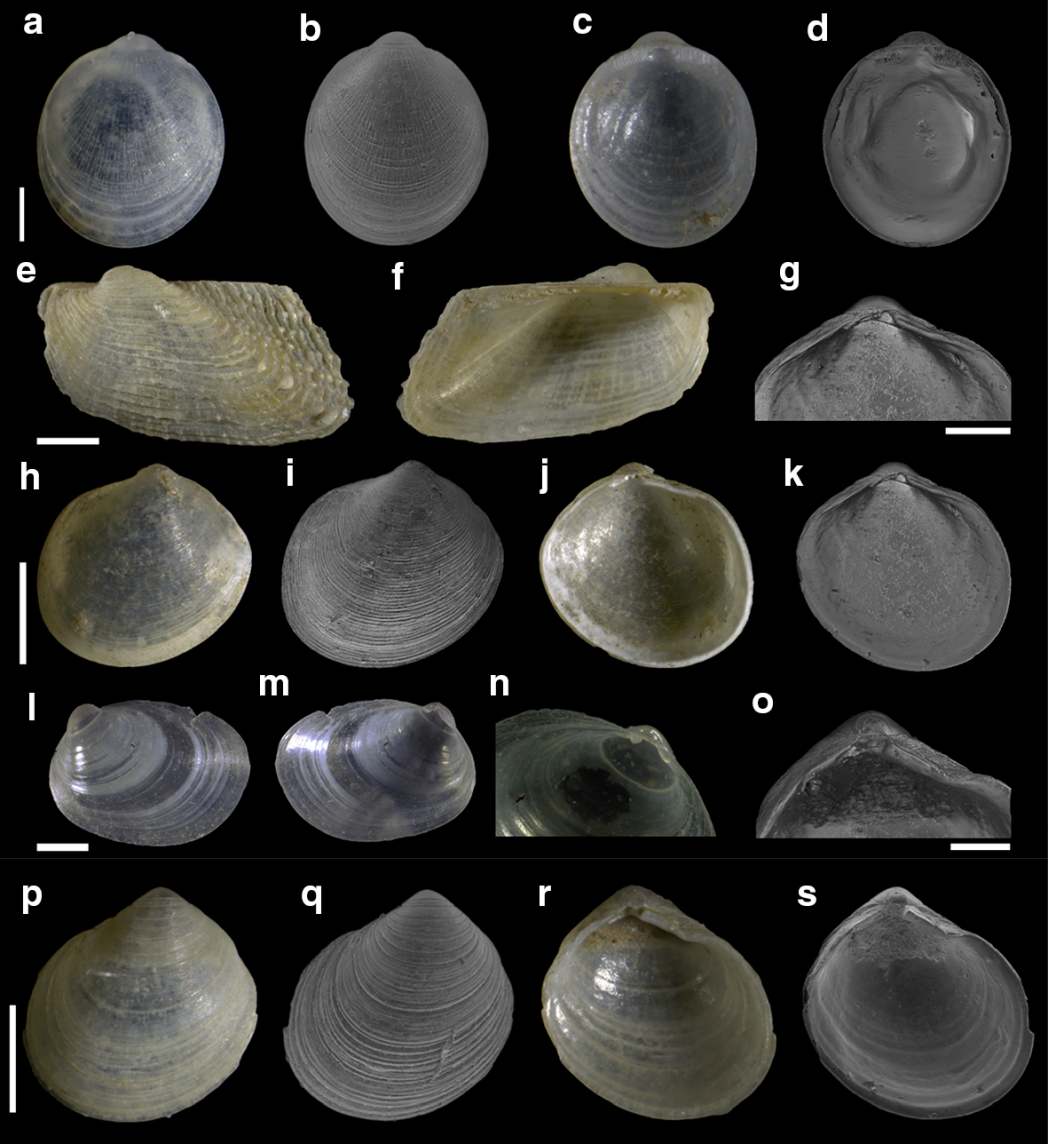
**a–d** *Crenella pellucida*, sample RC16. **e–f** *Asperarca magdalenae*, sample RC66. **g–k** *Kelliopsis jozinae*, sample RC16. **l–n** *Montacuta goudi*, sample TG80. **o–s** cf. *Draculamya porobranchiata*, sample RC16. Scale bars: a–d, g, l–m, o: 0.2 mm; e–f, h–k, p–s: 0.5 mm

Fourty-three species are here recorded for the first time for Israel: *Hanleya hanleyi*, *Emarginula adriatica*, *E. tenera*, *Petalopoma elisabettae*, *Epitonium tryoni* (Fig. 4a–d), *Cheirodonta pallescens*, *Marshallora adversa*, *Metaxia* sp., *Pogonodon pseudocanaricus*, *Similiphora similior*, *Cerithiopsis pulchresculpta* (Fig. 4e, f, j), *C. scalaris* (Fig. 4g, h, k), *Krachia cylindrata* (Fig. 4i, l–n), *Alvania mamillata*, *Setia amabilis*, *Granulina melitensis* (Fig. 5a–c), *Mitrella coccinea* (Fig. 5e–g), *Mitrella svelta* (Fig. 5d, h), *Fusinus buzzurroi*, *Haedropleura secalina*, *Mathilda bieleri* (Fig. 5i–j), *Graphis albida* (Fig. 6a–e), *Dondice banyulensis* (Fig. 2d), *Tylodina perversa* (Fig. 7i–k), *Notodiaphana atlantica* (Fig. 5k, l), *Pyrunculus hoernesi*, *Philine punctata* (Fig. 7h, l), *Odostomella bicincta* (Fig. 6f–h), *Odostomia sicula*, *O. turrita* (Fig. 6i–m), *Parthenina penchynati* (Fig. 7a–d), *Pyrgulina stefanisi* (Fig. 7e–g), *Tibersyrnola unifasciata*, *Crenella pellucida* (Fig. 8a–d), *Gregariella semigranata*, *Anadara corbuloides*, *Asperarca magdalenae* (Fig. 7e, f), *Parvicardium scabrum*, cf. *Draculamya porobranchiata* (Fig. 8o–s), *Kelliopsis jozinae* (Fig. 8g–k), *Montacuta goudi* (Fig. 8l–n), *Globivenus effossa* and *Pitar mediterraneus*.

The bivalve we identified as “cf. *Draculamya porobranchiata*” (Fig. 8o–s) is particularly intriguing. This species has been described from the North-East Atlantic Ocean at bathyal depths and belongs to a peculiar ectoparasitic genus that pierces unidentified hosts and feeds on fluids (Oliver and Lützen 2011). We found it in the reef habitat only. If confirmed, this would be the first record of this species from the Mediterranean Sea.

We also record four (2%) non-indigenous species represented by only seven (0.5%) specimens: *Melanella* sp. and *Parvioris* sp. collected alive only and whose taxonomic status will be discussed elsewhere, and *Turbonilla cangeyrani* and *Septifer cumingii* collected dead only.

## Discussion

### Biodiversity novelty in the Israeli mesophotic zone

The mesophotic molluscan diversity of Israel is severely underestimated. Notwithstanding we recorded 131 and 87 species from the hard and soft substrate, respectively, and 43 (25%) species were first records for Israel, diversity estimators suggested that the real diversity may be ~ 50% larger. This is probably the consequence of research mostly focused on shallower waters, difficulties in accessing these habitats, and taxonomic challenges.

Research on coastal benthic biodiversity in Israel was mostly conducted in the 1960s and 1970s, in the context of the 5-year Hebrew University—Smithsonian Institution Joint Program (1967–1972) focused on the Lessepsian invasion (Por et al. 1972) or of other large-scale surveys of benthic assemblages (e.g. Gilat 1964; Galil and Lewinsohn 1981; Tom and Galil 1991). All these endeavours sampled mostly shallow subtidal soft substrates. Further broad-scale sampling has been conducted after the year 2000 in the framework of the National Monitoring Programme. Despite this programme enabled the detection of several non-indigenous species (e.g. Guarnieri et al. 2017), it again targeted mainly soft and hard substrates in shallow water.

Indeed, the mesophotic presents challenges for its sampling and exploration, especially on hard substrates, which can be surveyed most effectively by technical diving or ROVs. The one here described is just the second effort to survey Israeli mesophotic reefs, after the recent exploration of the sponge reefs off Herzlyia, central Israel, at 95–120 m (Idan et al. 2018).

Taxonomic challenges further contribute to diversity underestimation. A paradigmatic example is the hyper-diverse gastropod family Triphoridae (Albano et al. 2011), which until the late 1970s was considered to contain the single Mediterranean species “*Triphora perversa*” (Piani 1980), but subsequent taxonomic work proved that it is a complex of more than 10 species (Bouchet and Guillemot 1978; Bouchet 1985, 1996). The family was represented in our samples by seven species, five (71%) recorded here for the first time. Further similar cases are the highly diverse Cerithiopsidae and Pyramidellidae, here represented by 5 and 21 species, respectively, of which 3 (60%) and 7 (33%) are new records. A second set of taxonomic cases is related to past misidentifications. It is hard to believe that, e.g., *Alvania mamillata*, a common rissoid also in shallow waters, has escaped detection since the late nineteenth century. It might have been misidentified by previous authors for *A. cimex*, from which it has been clearly separated only in the late 1980s (Verduin 1986; Amati et al. 2017). A last case is the one of recently described species belonging to poorly known groups such as *Petalopoma elisabettae*, *Granulina melitensis*, *Fusinus buzzurroi*, *Mathilda bieleri*, *Notodiaphana atlantica*, *Asperarca magdalenae* and cf. *Draculamya porobranchiata*, all described in the last ~ 20 years, a time that has seen a shrinking of the molluscan taxonomists’ community in Israel as well as an increasing focus on recording Lessepsian species.

As a last remark, we detected only four non-indigenous species, just 2% of the total species richness and 0.5% of the abundance, a result in stark contrast with the dominance of non-indigenous molluscs and other organisms in the shallow subtidal (Edelist et al. 2013; Rilov et al. 2018). Additionally, several native species were represented by large-sized adults, again in contrast with the shallow subtidal, where most species were represented by juveniles which may not reach the reproductive size (Albano et al., results under review). These results suggest that, in Israel, the mesophotic zone still hosts healthy native assemblages, as also noted by Idan et al. (2018). These assemblages lie at much lower temperatures, below 20 °C year around, than the shallow subtidal, where summer temperatures exceed 30 °C (analysis of the GLOBAL_ANALYSIS_FORECAST_PHY_001_024 dataset at https://marine.copernicus.eu/) and may work as climatic refugia because they are less exposed to thermal anomalies and related mass-mortality events (Cerrano et al. 2019). Therefore, they deserve strong conservation measures to protect their diversity, especially in areas like the easternmost Mediterranean Sea where climate warming and biological invasions are profoundly transforming the shallow shelf (Rilov et al. 2020).

### Is biodiversity underestimation a broader pattern in the Eastern Mediterranean?

A declining west to east native diversity gradient is reported for the Mediterranean Sea (Tortonese 1951; Coll et al. 2010) as a consequence of its geologic history and the west to east variation in environmental factors (eastward increase in temperature and salinity, and decrease in nutrients in particular) (Sabelli and Taviani 2014). However, part of the reported lower diversity in the Eastern Mediterranean may be attributed to insufficient sampling: every time a taxonomic group is thoroughly studied, a remarkable number of previously unreported native species is recorded (e.g. Morri et al. 2009; Idan et al. 2018; Crocetta et al. 2020; Achilleos et al. 2020; Castelló et al. 2020). Our results are no exception: the 43 newly reported species constitute an increase of 7% in relation to the 624 native molluscs previously recorded from Israel. Importantly, only 5 of these new records have been reported in recent surveys of the nearby Lebanon (Crocetta et al. 2013, 2014, 2020), suggesting that this result is robust over spatial scales broader than our study area.

Natural native biodiversity gradients are, however, being disrupted by the disappearance of native species from the warmest sectors of the Mediterranean Sea (Rilov 2016, Albano et al., results under review). Additionally, the massive entrance of non-indigenous species via the Suez Canal is further profoundly modifying assemblages (Galil 2009; Zenetos et al. 2012; Nunes et al. 2014; Rilov 2016). Proper understanding of the taxonomic and functional changes that these modifications are entailing requires the availability of data on pre-impact conditions. Due to the speed at which such changes are occurring in the Eastern Mediterranean, a thorough basin-scale survey of native biodiversity is mandatory before it is irremediably lost (Peleg et al. 2019; Yeruham et al. 2019).

## Electronic supplementary material

Below is the link to the electronic supplementary material.

Supplementary material 1 (PDF 357 kb)

## Data Availability

Quantitative data have been deposited in OBIS database.
