# Supplementary material for: Low diversity or poorly explored? Mesophotic molluscs highlight undersampling in the Eastern Mediterranean
Source: Biodivers Conserv. 2020 Oct 12;29(14):4059–72. doi: 10.1007/s10531-020-02063-w (PMC7658090; doi:10.1007/s10531-020-02063-w)
Supplement: Supplementary file 1 — Supplementary material 1 (PDF 357 kb) [file 10531_2020_2063_MOESM1_ESM.pdf]

Low diversity or poorly explored? Mesophotic molluscs highlight undersampling in the Eastern Mediterranean

Electronic Supplementary Material

Paolo G. Albano[1] (0000-0001-9876-1024), Michele Azzarone[1] (0000-0003-0197-6817), Bruno Amati[2] (0000-0002-3515-9543), Cesare Bogi[3] (0000-0001-7954-9562), Bruno Sabelli[4] (0000-0003-4300-3263), Gil Rilov[5] (0000-0002-1334-4887)

[1] Department of Palaeontology, University of Vienna, Althanstrasse 14, 1090 Vienna, Austria

[2] Largo Giuseppe Veratti, 37/D, 00146 Roma, Italy

[3] Gruppo Malacologico Livornese, c/o Museo di Storia Naturale del Mediterraneo, via Roma 234, 57127 Livorno, Italy

[4] Museo di Zoologia dell'Università di Bologna, via Selmi 3, 40126 Bologna, Italy

[5] National Institute of Oceanography, Israel Oceanographic and Limnological Research (IOLR), Haifa 3108001, Israel

Corresponding author: P.G. Albano, pgalbano@gmail.com

Quantitative data

Table S1. Molluscs found at two mesophotic sites on hard (-92 m depth) and soft (-77/83 m depth) substrates off the coast of northern Israel (NIS: non-indigenous species).

Class	Family	Genus	Species	NIS	Hard substrate		Soft substrate		First record in Israel
					Living	Shells	Living	Shells	
Polyplacophora	Hanleyidae	*Hanleya*	*hanleyi* (Bean, 1844)	No	0	0	0	1	This work
Gastropoda	Fissurellidae	*Diodora*	*gibberula* (Lamarck, 1822)	No	0	1	0	0	(Haas 1937)
Gastropoda	Fissurellidae	*Emarginula*	*adriatica* O.G. Costa, 1830	No	2	5	0	0	This work
Gastropoda	Fissurellidae	*Emarginula*	*huzardii* (Payraudeau, 1826)	No	0	6	0	0	(Barash and Danin 1992)
Gastropoda	Fissurellidae	*Emarginula*	*tenera* Locard, 1891	No	0	2	0	0	This work
Gastropoda	Scissurellidae	*Scissurella*	*costata* d'Orbigny, 1824	No	0	15	0	1	(Barash and Danin 1992)
Gastropoda	Trochidae	*Jujubinus*	*exasperatus* (Pennant, 1777)	No	0	2	0	0	(Haas 1937)
Gastropoda	Trochidae	*Jujubinus*	*montagui* (Wood, 1828)	No	0	0	0	1	(Barash and Danin 1992)
Gastropoda	Trochidae	*Jujubinus*	*striatus* (Linnaeus, 1758)	No	0	1	0	0	(Mienis and Ben-David-Zaslow 2004)
Gastropoda	Calliostomatidae	*Calliostoma*	*conulus* (Linnaeus, 1758)	No	1	9	0	4	(Avnimelech and Boskovitz 1955)
Gastropoda	Turbinidae	*Bolma*	*rugosa* (Linnaeus, 1767)	No	0	0	0	1	(Haas 1937)

Class	Family	Genus	Species	NIS	Hard substrate		Soft substrate		First record in Israel
					Living	Shells	Living	Shells	
Gastropoda	Cerithiidae	*Bittium*	*latreillii* (Payraudeau, 1826)	No	11	127	0	11	(Mienis and Ben-David-Zaslow 2004)
Gastropoda	Cerithiidae	*Bittium*	reticulatum (da Costa, 1778)	No	0	24	0	0	(Hart 1891)
Gastropoda	Cerithiidae	*Bittium*	*submamillatum* (de Rayneval & Ponzi, 1854)	No	0	0	1	71	(Barash and Danin 1992)
Gastropoda	Siliquariidae	*Petalopoma*	*elisabettae* Schiaparelli, 2002	No	0	23	0	0	This work
Gastropoda	Siliquariidae	*Tenagodus*	*obtusus* (Schumacher, 1817)	No	0	7	0	2	(Haas 1937)
Gastropoda	Turritellidae	*Turritellinella*	*tricarinata* (Brocchi, 1814)	No	0	1	0	2	(Haas 1951)
Gastropoda	Turritellidae	*Turritella*	*turbona* Monterosato, 1877	No	7	63	0	4	(Comay et al. 2015)
Gastropoda	Epitoniidae	*Aclis*	*minor* (Brown, 1827)	No	0	0	1	0	(Haas 1951)
Gastropoda	Epitoniidae	*Epitonium*	*algerianum* (Weinkauff, 1866)	No	0	0	0	2	(Barash and Danin 1992)
Gastropoda	Epitoniidae	*Epitonium*	*muricatum* (Risso, 1826)	No	0	0	0	2	(Haas 1951)
Gastropoda	Epitoniidae	*Epitonium*	*tryoni* (de Boury, 1913)	No	0	0	0	1	This work
Gastropoda	Naticidae	*Naticarius*	*stercusmuscarum* (Gmelin, 1791)	No	0	0	0	2	(Aharoni 1934)
Gastropoda	Triphoridae	*Cheirodonta*	*pallescens* (Jeffreys, 1867)	No	0	5	0	1	This work
Gastropoda	Triphoridae	*Marshallora*	*adversa* (Montagu, 1803)	No	1	1	0	0	This work
Gastropoda	Triphoridae	*Metaxia*	sp.	No	0	1	0	0	This work
Gastropoda	Triphoridae	*Metaxia*	*metaxa* (Delle Chiaje, 1828)	No	0	2	0	0	(Haas 1951)
Gastropoda	Triphoridae	*Monophorus*	*perversus* (Linnaeus, 1758)	No	0	1	0	0	(Haas 1937)
Gastropoda	Triphoridae	*Ionthoglossa*	*pseudocanarica* (Bouchet, 1985)	No	1	7	0	0	This work
Gastropoda	Triphoridae	*Similiphora*	*similior* (Bouchet & Guillemot, 1978)	No	1	0	0	0	This work
Gastropoda	Cerithiopsidae	*Cerithiopsis*	*diadema* Monterosato, 1874	No	0	5	0	2	(Barash and Danin 1992)
Gastropoda	Cerithiopsidae	*Cerithiopsis*	*pulchresculpta* Cachia, Mifsud & Sammut, 2004	No	0	1	0	0	This work
Gastropoda	Cerithiopsidae	*Cerithiopsis*	*scalaris* Locard, 1892	No	0	1	0	1	This work
Gastropoda	Cerithiopsidae	*Dizoniopsis*	*concatenata* (Conti, 1864)	No	1	1	0	0	(Barash and Danin 1992)
Gastropoda	Cerithiopsidae	*Krachia*	*cylindrata* (Jeffreys, 1885)	No	1	2	0	0	This work
Gastropoda	Rissoidae	*Alvania*	*beani* (Hanley in Thorpe, 1844)	No	0	4	0	1	(Avnimelech and Boskovitz 1955)
Gastropoda	Rissoidae	*Alvania*	*mamillata* Risso, 1826	No	6	94	0	0	This work
Gastropoda	Rissoidae	*Alvania*	*testae* (Aradas & Maggiore, 1844)	No	0	3	0	0	(Haas 1951)
Gastropoda	Rissoidae	*Crisilla*	*semistriata* (Montagu, 1808)	No	0	1	0	0	(Pallary 1938)
Gastropoda	Rissoidae	*Obtusella*	*macilenta* (Monterosato, 1880)	No	0	3	0	10	(Mienis 1983)
Gastropoda	Rissoidae	*Pusillina*	*inconspicua* (Alder, 1844)	No	1	24	0	2	(van Aartsen et al. 1989)
Gastropoda	Rissoidae	*Setia*	*amabilis* (Locard, 1886)	No	0	3	0	0	This work
Gastropoda	Iravadiidae	*Hyala*	*vitrea* (Montagu, 1803)	No	0	0	0	2	(Barash and Danin 1992)
Gastropoda	Eulimidae	*Melanella*	sp.	Yes	2	0	0	0	This work
Gastropoda	Eulimidae	*Parvioris*	sp.	Yes	1	0	0	0	This work
Gastropoda	Eulimidae	*Nanobalcis*	*nana* (Monterosato, 1878)	No	0	8	0	0	(Mienis and Ben-David-Zaslow 2004)
Gastropoda	Eulimidae	*Sticteulima*	*jeffreysiana* (Brusina, 1869)	No	7	12	0	1	(van Aartsen et al. 1989)
Gastropoda	Cypraeidae	*Luria*	*lurida* (Linnaeus, 1758)	No	1	0	0	0	(Mienis and Ben-David-Zaslow 2004)
Gastropoda	Cypraeidae	*Naria*	*spurca* (Linnaeus, 1758)	No	0	0	0	1	(Aharoni 1934)
Gastropoda	Aporrhaidae	*Aporrhais*	*pespelecani* (Linnaeus, 1758)	No	0	0	0	4	(Pallary 1938)
Gastropoda	Marginellidae	*Volvarina*	*mitrella* (Risso, 1826)	No	0	0	0	1	(Haas 1937)

Class	Family	Genus	Species	NIS	Hard substrate		Soft substrate		First record in Israel
					Living	Shells	Living	Shells	
Gastropoda	Granulinidae	*Granulina*	*melitensis* Smriglio, Mariottini & Rufini, 1998	No	1	13	0	3	This work
Gastropoda	Buccinidae	*Euthria*	*cornea* (Linnaeus, 1758)	No	0	0	0	1	(Aharoni 1934)
Gastropoda	Colubrariidae	*Cumia*	*reticulata* (Blainville, 1829)	No	0	10	0	0	(Haas 1937)
Gastropoda	Columbellidae	*Mitrella*	*coccinea* (Philippi, 1836)	No	2	18	0	0	This work
Gastropoda	Columbellidae	*Mitrella*	*minor* (Scacchi, 1836)	No	0	0	0	3	(Haas 1951)
Gastropoda	Columbellidae	*Mitrella*	*svelta* Kobelt, 1889	No	1	3	0	0	This work
Gastropoda	Fasciolariidae	*Fusinus*	*buzzurroi* Prkić & Russo, 2008	No	2	8	0	1	This work
Gastropoda	Nassariidae	*Nassarius*	*turulosus* (Risso, 1826)	No	0	7	0	2	(Haas 1951)
Gastropoda	Nassariidae	*Tritia*	*incrassata* (Strøm, 1768)	No	0	1	0	1	(Hart 1891)
Gastropoda	Nassariidae	*Tritia*	*pygmaea* (Lamarck, 1822)	No	0	0	0	24	(Nordsieck 1972)
Gastropoda	Pisaniidae	*Enginella*	*leucozona* (Philippi, 1844)	No	2	13	0	0	(Hart 1891)
Gastropoda	Muricidae	*Coralliophila*	*meyendorffii* (Calcara, 1845)	No	0	2	0	0	(Barash and Danin 1992)
Gastropoda	Muricidae	*Muricopsis*	*cristata* (Brocchi, 1814)	No	0	2	0	0	(Hart 1891)
Gastropoda	Muricidae	*Typhinellus*	*labiatus* (de Cristofori & Jan, 1832)	No	0	0	0	1	(Haas 1937)
Gastropoda	Borsoniidae	*Drilliola*	*loprestiana* (Calcara, 1841)	No	0	0	0	1	(Haas 1951)
Gastropoda	Clathurellidae	*Comarmondia*	*gracilis* (Montagu, 1803)	No	0	0	0	1	(Haas 1951)
Gastropoda	Drilliidae	*Crassopleura*	*maravignae* (Bivona Ant. in Bivona And., 1838)	No	5	7	0	0	(Haas 1951)
Gastropoda	Horaiclavidae	*Haedropleura*	*secalina* (Philippi, 1844)	No	0	1	0	0	This work
Gastropoda	Mangeliidae	*Bela*	*zenetouae* (van Aartsen, 1988)	No	0	0	0	1	(van Aartsen 1988)
Gastropoda	Mangeliidae	*Mangelia*	*costata* (Pennant, 1777)	No	0	2	0	0	(Barash and Danin 1992)
Gastropoda	Mangeliidae	*Mangelia*	*costulata* Risso, 1826	No	0	1	0	0	(Nordsieck 1972)
Gastropoda	Mangeliidae	*Sorgenfreispira*	*brachystoma* (Philippi, 1844)	No	0	0	0	2	(Haas 1951)
Gastropoda	Mitromorphidae	*Mitromorpha*	*columbellaria* (Scacchi, 1836)	No	1	10	0	0	(Haas 1951)
Gastropoda	Raphitomidae	*Clathromangelia*	*granum* (Philippi, 1844)	No	0	5	0	0	(Barash and Danin 1992)
Gastropoda	Raphitomidae	*Raphitoma*	*leufroyi* (Michaud, 1828)	No	0	1	0	0	(Barash and Danin 1992)
Gastropoda	Raphitomidae	*Raphitoma*	*linearis* (Montagu, 1803)	No	0	1	0	0	(Barash and Danin 1992)
Gastropoda	Raphitomidae	*Raphitoma*	*pseudohystrix* (Sykes, 1906)	No	0	3	0	0	(Barash and Danin 1992)
Gastropoda	Architectonicidae	*Pseudomalaxis*	*zancleus* (Philippi, 1844)	No	0	2	0	0	(Barash and Danin 1992)
Gastropoda	Architectonicidae	*Solatisonax*	*alleryi* (Seguenza G., 1876)	No	0	7	0	1	(Haas 1951)
Gastropoda	Mathildidae	*Mathilda*	*bieleri* Smriglio & Mariottini, 2007	No	0	1	0	0	This work
Gastropoda	Cimidae	*Graphis*	*albida* (Kanmacher, 1798)	No	0	0	0	1	This work
Gastropoda	Pleurobranchidae	*Berthella*	*plumula* (Montagu, 1803)	No	0	1	0	0	(O'Donoghue and White 1940)
Gastropoda	Ringiculidae	*Ringicula*	*auriculata* (Ménard de la Groye, 1811)	No	0	0	0	16	(Haas 1937)
Gastropoda	Chromodorididae	*Felimare*	*picta* (Philippi, 1836)	No	1	0	0	0	(Barash and Danin 1971)
Gastropoda	Myrrhinidae	*Dondice*	*banyulensis* Portmann & Sandmeier, 1960	No	1	0	0	0	This work
Gastropoda	Tylodinidae	*Tylodina*	*perversa* (Gmelin, 1791)	No	0	2	0	0	This work
Gastropoda	Notodiaphanidae	*Notodiaphana*	*atlantica* Ortea, Moro & Espinosa, 2013	No	0	2	0	0	This work
Gastropoda	Retusidae	*Pyrunculus*	*hoernesi* (Weinkauff, 1866)	No	0	4	0	0	This work
Gastropoda	Retusidae	*Retusa*	*minutissima* Thiele, 1931	No	0	0	0	1	(van Aartsen et al. 1989)

Class	Family	Genus	Species	NIS	Hard substrate		Soft substrate		First record in Israel
					Living	Shells	Living	Shells	
Gastropoda	Philinidae	*Philine*	*punctata* (J. Adams, 1800)	No	0	0	0	1	This work
Gastropoda	Pyramidellidae	*Eulimella*	*acicula* (Philippi, 1836)	No	0	1	0	2	(Haas 1951)
Gastropoda	Pyramidellidae	*Eulimella*	*scillae* (Scacchi, 1836)	No	0	0	0	2	(Barash and Danin 1992)
Gastropoda	Pyramidellidae	*Eulimella*	*ventricosa* (Forbes, 1844)	No	1	8	0	0	(Barash and Danin 1992)
Gastropoda	Pyramidellidae	*Megastomia*	*conoidea* (Brocchi, 1814)	No	0	2	0	10	(Haas 1937)
Gastropoda	Pyramidellidae	*Odostomella*	*bicincta* (Tiberi, 1868)	No	0	8	0	1	This work
Gastropoda	Pyramidellidae	*Odostomella*	*doliolum* (Philippi, 1844)	No	2	0	0	0	(Barash and Danin 1992)
Gastropoda	Pyramidellidae	*Odostomia*	*acuta* Jeffreys, 1848	No	0	1	0	0	(Barash and Danin 1992)
Gastropoda	Pyramidellidae	*Odostomia*	*lukisii* Jeffreys, 1859	No	0	1	0	0	(Nordsieck 1972)
Gastropoda	Pyramidellidae	*Odostomia*	*sicula* sensu Auctores non Philippi, 1851	No	0	1	0	0	This work
Gastropoda	Pyramidellidae	*Odostomia*	*turrita* Hanley, 1844	No	0	3	0	0	This work
Gastropoda	Pyramidellidae	*Odostomia*	*unidentata* (Montagu, 1803)	No	0	5	0	0	(Haas 1951)
Gastropoda	Pyramidellidae	*Parthenina*	*Interstincta* (J. Adams, 1797)	No	0	0	0	4	(Nordsieck 1972)
Gastropoda	Pyramidellidae	*Parthenina*	*penchynati* (Bucquoy, Dautzenberg & Dollfus, 1883)	No	0	5	0	4	This work
Gastropoda	Pyramidellidae	*Parthenina*	*suturalis* (Philippi, 1844)	No	0	3	0	0	(van Aartsen et al. 1989)
Gastropoda	Pyramidellidae	*Pyrgulina*	*stefanisi* (Jeffreys, 1869)	No	0	0	0	2	This work
Gastropoda	Pyramidellidae	*Tibersyrnola*	*unifasciata* (Forbes, 1844)	No	0	3	0	0	This work
Gastropoda	Pyramidellidae	*Tragula*	*fenestrata* (Jeffreys, 1848)	No	0	1	0	0	(Barash and Danin 1992)
Gastropoda	Pyramidellidae	*Turbonilla*	*cangeyrani* Ovalis & Mifsud, 2017	Yes	0	1	0	0	This work
Gastropoda	Pyramidellidae	*Turbonilla*	*gradata* Bucquoy, Dautzenberg & Dollfus, 1883	No	0	1	0	0	(Haas 1951)
Gastropoda	Pyramidellidae	*Turbonilla*	*lactea* (Linnaeus, 1758)	No	0	0	0	2	(Haas 1951)
Gastropoda	Pyramidellidae	*Turbonilla*	*pusilla* (Philippi, 1844)	No	0	4	0	0	(van Aartsen et al. 1989)
Gastropoda	Amathinidae	*Clathrella*	*clathrata* (Philippi, 1844)	No	0	2	0	0	(Barash and Danin 1992)
Bivalvia	Nuculidae	*Nucula*	*nitidosa* Winckworth, 1930	No	0	1	0	7	(Barash and Danin 1992)
Bivalvia	Nuculidae	*Nucula*	*sulcata* Bronn, 1831	No	0	1	0	3	(Haas 1951)
Bivalvia	Nuculanidae	*Lembulus*	*pella* (Linnaeus, 1758)	No	0	0	0	2	(Haas 1937)
Bivalvia	Nuculanidae	*Saccella*	*commutata* (Philippi, 1844)	No	0	12	0	7	(Haas 1951)
Bivalvia	Mytilidae	*Crenella*	*arenaria* Monterosato, 1875 ex H. Martin, ms.	No	0	1	1	0	(Mienis and Rittner 2014)
Bivalvia	Mytilidae	*Crenella*	*pellucida* (Jeffreys, 1859)	No	0	1	0	0	This work
Bivalvia	Mytilidae	*Dacrydium*	*hyalinum* (Monterosato, 1875)	No	4	6	0	0	(Barash and Danin 1992)
Bivalvia	Mytilidae	*Gregariella*	*semigranata* (Reeve, 1858)	No	56	102	2	1	This work
Bivalvia	Mytilidae	*Modiolula*	*phaseolina* (Philippi, 1844)	No	3	6	0	1	(van Aartsen et al. 1989)
Bivalvia	Mytilidae	*Musculus*	*subpictus* (Cantraine, 1835)	No	1	3	0	4	(Barash and Danin 1992)
Bivalvia	Mytilidae	*Septifer*	*cumingii* Récluz, 1848	Yes	0	3	0	0	This work
Bivalvia	Arcidae	*Acar*	*clathrata* (Defrance, 1816)	No	0	1	0	0	(Moshkovitz 1963)
Bivalvia	Arcidae	*Anadara*	*corbuloides* (Monterosato, 1881)	No	0	1	0	23	This work
Bivalvia	Arcidae	*Arca*	*tetragona* Poli, 1795	No	0	1	0	0	(Barash and Danin 1992)

Class	Family	Genus	Species	NIS	Hard substrate		Soft substrate		First record in Israel
					Living	Shells	Living	Shells	
Bivalvia	Arcidae	*Asperarca*	*magdalenae* La Perna, 1998	No	0	1	0	0	This work
Bivalvia	Arcidae	*Barbatia*	*barbata* (Linnaeus, 1758)	No	0	0	0	1	(Hart 1891)
Bivalvia	Glycymerididae	*Glycymeris*	*glycymeris* (Linnaeus, 1758)	No	0	2	0	0	(Weinkauff 1867)
Bivalvia	Noetiidae	*Striarca*	*lactea* (Linnaeus, 1758)	No	1	27	1	24	(Haas 1937)
Bivalvia	Anomiidae	*Anomia*	*ephippium* Linnaeus, 1758	No	0	0	0	1	(Haas 1937)
Bivalvia	Pectinidae	*Aequipecten*	*opercularis* (Linnaeus, 1758)	No	0	0	0	1	(Haas 1951)
Bivalvia	Pectinidae	*Karnekampia*	*sulcata* (Müller, 1776)	No	0	1	0	0	(Huber 2010)
Bivalvia	Pectinidae	*Mimachlamys*	*varia* (Linnaeus, 1758)	No	0	1	0	1	(Hart 1891)
Bivalvia	Pectinidae	*Palliolum*	*incomparabile* (Risso, 1826)	No	0	0	0	1	(van Aartsen et al. 1989)
Bivalvia	Pectinidae	*Talochlamys*	*multistriata* (Poli, 1795)	No	0	1	0	0	(Barash and Danin 1992)
Bivalvia	Propeamussiidae	*Similipecten*	*similis* (Laskey, 1811)	No	0	1	0	22	(Barash and Danin 1992)
Bivalvia	Limidae	*Lima*	*lima* (Linnaeus, 1758)	No	0	1	0	0	(Aharoni 1934)
Bivalvia	Limidae	*Limatula*	*gwyni* (Sykes, 1903)	No	0	1	0	0	(van Aartsen et al. 1989)
Bivalvia	Lucinidae	*Loripinus*	*lragilis* (Philippi, 1836)	No	0	1	0	0	(Barash and Danin 1992)
Bivalvia	Carditidae	*Centrocardita*	*aculeata* (Poli, 1795)	No	0	5	0	13	(Haas 1951)
Bivalvia	Carditidae	*Glans*	*trapezia* (Linnaeus, 1767)	No	0	1	0	0	(Haas 1937)
Bivalvia	Trapezidae	*Coralliophaga*	*lithophagella* (Lamarck, 1819)	No	0	1	0	0	(Barash and Danin 1992)
Bivalvia	Cardiidae	*Papillicardium*	*papillosum* (Poli, 1791)	No	0	1	0	7	(Haas 1951)
Bivalvia	Cardiidae	*Parvicardium*	*exiguum* (Gmelin, 1791)	No	0	2	0	0	(Lipkin and Safriel 1971)
Bivalvia	Cardiidae	*Parvicardium*	*minimum* (Philippi, 1836)	No	0	3	0	13	(Moshkovitz 1963)
Bivalvia	Cardiidae	*Parvicardium*	*scabrum* (Philippi, 1844)	No	0	0	0	16	This work
Bivalvia	Lasaeidae	cf. *Draculamya*	*porobranchiata* P.G. Oliver & Lützen, 2011	No	1	1	0	0	This work
Bivalvia	Lasaeidae	*Kelliopsis*	*jozinae* van Aartsen & Carrozza, 1997	No	1	2	0	1	This work
Bivalvia	Lasaeidae	*Kurtiella*	*bidentata* (Montagu, 1803)	No	0	2	0	8	(Haas 1951)
Bivalvia	Lasaeidae	*Montacuta*	*goudi* van Aartsen, 1997	No	0	0	0	1	This work
Bivalvia	Lasaeidae	*Tellimya*	*ferruginosa* (Montagu, 1808)	No	0	0	0	1	(Barash and Danin 1992)
Bivalvia	Kelliellidae	*Kelliella*	*miliaris* (Philippi, 1844)	No	1	4	0	0	Janssen (1989)
Bivalvia	Tellinidae	*Moerella*	*distorta* (Poli, 1791)	No	0	0	0	1	(Haas 1951)
Bivalvia	Semelidae	*Abra*	*alba* (W. Wood, 1802)	No	0	2	0	10	(Haas 1951)
Bivalvia	Ungulinidae	*Diplodonta*	*brocchii* (Deshayes, 1850)	No	0	1	0	0	(Barash and Danin 1992)
Bivalvia	Veneridae	*Dosinia*	*lupinus* (Linnaeus, 1758)	No	0	1	0	1	(Haas 1951)
Bivalvia	Veneridae	*Globivenus*	*effossa* (Philippi, 1836)	No	0	1	0	1	This work
Bivalvia	Veneridae	*Gouldia*	*minima* (Montagu, 1803)	No	0	1	0	1	(Haas 1951)
Bivalvia	Veneridae	*Pitar*	*mediterraneus* (Aradas & Benoit, 1872)	No	0	3	0	30	This work
Bivalvia	Veneridae	*Timoclea*	*ovata* (Pennant, 1777)	No	0	4	0	30	(Haas 1951)
Bivalvia	Veneridae	*Venus*	*casina* Linnaeus, 1758	No	0	0	0	1	(Barash and Danin 1992)
Bivalvia	Myidae	*Sphenia*	*binghami* Turton, 1822	No	3	4	0	0	(Barash and Danin 1992)
Bivalvia	Hiatellidae	*Hiatella*	*arctica* (Linnaeus, 1767)	No	0	4	0	4	(Haas 1951)
Bivalvia	Hiatellidae	*Hiatella*	*rugosa* (Linnaeus, 1767)	No	0	1	0	0	(Barash and Danin 1992)
Bivalvia	Cuspidariidae	*Cardiomya*	*costellata* (Deshayes, 1835)	No	0	1	0	1	(Haas 1951)
Bivalvia	Cuspidariidae	*Cuspidaria*	*rostrata* (Spengler, 1793)	No	0	1	0	0	(Haas 1951)

Class	Family	Genus	Species	NIS	Hard substrate		Soft substrate		First record in Israel
					Living	Shells	Living	Shells	
Scaphopoda	Gadilidae	*Dischides*	*politus* (S. Wood, 1842)	No	0	0	0	7	(Haas 1951)
Scaphopoda	Dentaliidae	*Antalis*	*inaequicostata* (Dautzenberg, 1891)	No	0	0	0	11	(Caprotti 1965)
Scaphopoda	Entalinidae	*Entalina*	*tetragona* (Brocchi, 1814)	No	0	1	0	0	(Haas 1951)

References

Aharoni J (1934) From Ashqelon to Rubin. Teva va-Aretz 2:472–476

Avnimelech MA, Boskovitz V (1955) Some problems of the present distribution of molluscan shells on the Mediterranean coast of Israel. Bulletin of the Research Council of Israel 5B:178–188

Barash A, Danin Z (1992) Annotated list of Mediterranean molluscs of Israel and Sinai. The Israel Academy of Sciences and Humanities, Jerusalem

Barash A, Danin Z (1971) Opisthobranchia (Mollusca) from the Mediterranean waters of Israel. Israel Journal of Zoology 20:151–200

Caprotti E (1965) Notes on the Mediterranean Dentaliidae (Studies on Scaphopoda, V). Atti della Società Italiana di Scienze Naturali e del Museo Civico di Storia Naturale di Milano 104:339–354

Comay O, Edelman-Furstenberg Y, Ben-Ami F (2015) Patterns in molluscan death assemblages along the Israeli Mediterranean continental shelf. Quaternary International 390:21–28. https://doi.org/10.1016/j.quaint.2015.05.004

Haas G (1937) Mollusca Marina. In: Bodenheimer F.S. (eds). In: Prodromus Faunae Palestinae. Essai sur les éléments zoogéographiques et historiques du sud-ouest du sous-régne paléarctique. Imprimerie de l’Institut Français d’Archéologie Orientale, Cairo

Haas G (1951) Preliminary report on the Molluscs of the Palestine coastal shelf. Bulletin Sea Fisheries Research Station Israel 1:1–20

Hart HC (1891) Some account of the fauna and flora of Sinai, Petra and Wâdy ‘Arabah. The Committee of the Palestine Exploration Fund, London

Huber M (2010) Compendium of bivalves. ConchBooks, Hackenheim

Lipkin Y, Safriel U (1971) Intertidal zonation on rocky shores at Mikhmoret (Mediterranean, Israel). Journal of Ecology 59:1–30

Mienis HK (1983) Recent publications concerning our region, 11. Levantina 45:532–534

Mienis HK, Ben-David-Zaslow R (2004) A preliminary list of the marine molluscs of the National Park and Nature reserve of Akhziv-Rosh Haniqra. Triton 10:13–37

Mienis HK, Rittner O (2014) Finds of new or little known molluscs from the Levantine Sea off Israel 1. Triton 30:5–8

Moshkovitz S (1963) The Mollusca in the upper part of the “Sakiebeds” (Upper Neogene-Lower Pleistocene) in the Central Coastal Plain of Israel. Israel Journal of Earth Sciences 12:97–146

Nordsieck F (1972) Marine Gastropoden aus der Shiqmona-Bucht in Israel. Archive für Molluskenkunde 102:227–245

O’Donoghue CH, White KM (1940) A collection of marine molluscs, mainly opisthobranchs, from Palestine. Proceedings of the Malacological Society of London 24:92–96

Pallary P (1938) Les mollusques marins de la Syrie. Journal de Conchyliologie 82:5–58

van Aartsen JJ (1988) Molluschi d’Europa: note su specie poco conosciute. XII. *Bela menkhorsti* nom. nov. = *Pleurotoma nana* Scacchi, 1836 non Deshayes, 1835 e *Fehria* (nov. gen.) *zenetouae* n. sp.. La Conchiglia 20:30–31

van Aartsen JJ, Barash A, Carrozza F (1989) Addition to the knowledge of the Mediterranean Mollusca of Israel and Sinai. Bollettino Malacologico 25:63–76

Weinkauff HC (1867) Die Conchylien des Mittelmeeres, ihre geographische und geologisches Verbreitung. Vol. 1., Mollusca Acephalia. T. Fischer, Cassel
